# Correlation study and full management of metabolic syndrome and sperm DNA fragmentation index, a regional multicentre prospective long term follow-up study from Anhui, China

**DOI:** 10.3389/fcell.2025.1586069

**Published:** 2025-05-02

**Authors:** Dongsheng Ma, Jianhong Xi

**Affiliations:** ^1^ Department of Reproductive Medicine, The People’s Hospital Bozhou, Bozhou, China; ^2^ Department of Urology, The First Affiliated Hospital of Xinjiang Medical University, Urumqi, China

**Keywords:** metabolic syndrome, sperm DNA fragmentation index, male infertility, obesity, metabolic components

## Abstract

**Objective:**

To investigate the correlation between Metabolic syndrome (Mets) and Sperm DNA fragmentation index (DFI) in men of reproductive age, and to summarise the Mets and metabolic component health management model in men.

**Methods:**

The Male Reproductive Health Follow-up Database in Bozhou City, Anhui Province, China (2020–2024) included in the study 1,008 outpatient cases of men with reproductive age,in which normal sperm DFI was the Control group (n = 858) and abnormal DFI was the Observation group (n = 150), and the general data, metabolic endocrine related indicators, and indicators related to fertility assessment were analysed in both groups, and fertility and metabolic indicators were followed up. Spearman rank correlation coefficient was used for correlation analysis, segmented package for threshold analysis, Bootstrap sampling method and Bayesian method for mediation effect test analysis. Univariate-multivariate logistic regression analyses were performed to build a predictive model using R Programming Language (4.42), and to plot the Nomogram, Calibration Curve, Decision Curve Analysis (DCA) Curve, and Clinical impact curve (CIC) to assess the consistency between the predicted probability of the model and the actual occurrence probability, as well as to evaluate the practicality and applicability in clinical decision-making.

**Results:**

Intergroup comparison between the observation and control groups in this study showed no statistical difference between the two groups in terms of baseline information and fertility assessment (*P* > 0.05). However, there was statistical difference between the two groups in MetS and metabolic scores (*P* < 0.001). One-way ANOVA showed a statistically significant difference between DFI and MetS scores (*P* = 0.021), and two-way comparisons showed a statistically significant difference between the groups with 0–4 points (*P* < 0.05). There was a moderate-strength positive correlation between metabolic score and DFI by Spearman’s correlation analysis (r = 0.475, *P* < 0.001). Overall, DFI and MetS were positively associated [OR (95%CI):1.09 (1.07–1.11)] when DFI< 32.26 [OR (95%CI): 1.15 (1.12–1.19)]. In the overall analysis, the association between MetS and adverse maternity outcomes was statistically significant (OR = 1.50, 95% CI: 1.01–2.22, *P* = 0.045). In the sperm DFI subgroup, the association of MetS with adverse maternity outcomes was significant in both DFI ≤15 and DFI >30 (15: OR = 2.51, 95%CI: 1.01–6.22, *P* = 0.047; >30:OR = 2.94, 95%CI: 1.19–7.22, *P* = 0.019), and subgroup analyses of age showed significant association between MetS and adverse maternity outcomes in age >30 years (OR = 1.94, 95% CI: 1.13–3.33, *P* = 0.016). The results of the mediated analysis pathway showed that obesity and hyperlipidaemia lead to sperm DFI abnormalities, which indirectly contribute to adverse maternity outcomes, but it has not been proven that sperm DFI abnormalities contribute to the occurrence of adverse maternity outcomes. The results of multifactorial logistic regression analysis showed that varicocele (OR = 1.975), obesity (OR = 2.296), hyperlipidaemia (OR = 2.422), and Low-HDL (OR = 3.654) were the independent risk factors for abnormal sperm DFI. And effective interventions for the group with abnormal sperm DFI could significantly reduce sperm DFI values and metabolic scores (*P* < 0.001). The predictive model has been validated to show positive predictive efficacy and clinical benefit.

**Conclusion:**

MetS may lead to abnormal sperm DNA fragmentation indices, which in turn suggests that abnormal sperm DFI due to MetS may be a risk factor for male infertility and spousal adverse maternity, and that effective interventions to reduce sperm DFI values and metabolic scores are necessary and urgent. This study is part of the China Anhui Regional Male Fertility Survey Phase I (2020–2024).

## Highlights

• This study is a regional multicentre study with sufficient sample size and long follow-up time, which can reflect the correlation between MetS and sperm DFI. However, because most of the study participants were outpatient clinics, there were unavoidable selection bias and baseline differences, and the data were based on a male population from the northern part of Anhui Province, China, which has certain differences in dietary structure, living habits and occupational exposure, and is slightly under-representative. Therefore, it is hoped that subsequent studies will include a diverse group of men. This study truly and objectively reflects the results of male fertility assessment and long-term follow-up in northern Anhui, China, and also promotes the multiple models of clinical diagnosis and treatment, specialist care, and out-of-hospital continuous health management in the field of male reproductive health, so as to provide theoretical support for the standardised assessment of fertility, long term follow-up, and continuous monitoring of fertility for the male population of childbearing age, which is not confined to men with the metabolic syndrome.
• This study provides further insight into the overall correlation between MetS and male fertility assessment (not limited to sperm DFI), but the assessment of multiple cumulative factors of MetS may be inappropriate for the study of male fertility, and the combining of multiple subgroups of the metabolic syndrome into a single variable may confound or obscure the cumulative effects and interactions of these different metabolic components on male fertility that result in inaccurate conclusions and confusing concepts. Follow-up studies will progressively adopt a larger multicentre sample size to complete a stratified evaluation of the weighting of the different metabolic subgroups, as well as the impact on sperm DFI and subsequent potential fertility risks in men. This will help researchers to better understand the cumulative effects of different metabolic subgroups on male fertility, so that these risky metabolic factors can be targeted for prevention or reduction.
• The present study, despite the inclusion of study subjects in an attempt to control for a variety of known risk factors for male fertility (chromosomal structural abnormalities in men and their spouses, familial dominant or invisible genetic disorders, disorders with a genetic predisposition, and so on), could not exclude or potentially or undetectably confounding factors. The study of fertility risk correlations between metabolic syndromes and single-factor men with different sperm DFI using different male sperm DFI cut-off values complicates the study of different subgroups but also more accurately reflects the differences between different male sperm DFI subgroups.
• In this study, there were irregularities in the follow-up of some patients during the follow-up process, specifically in the follow-up time and follow-up data errors, the later stages of the study to improve and standardise the process of patient follow-up, so that the recorded data to minimize the errors, and at the same time, in the whole process of the study, selecting the links of segment quality control and data quality control and other ways. Some blood samples and all semen samples were tested by homogenised accredited institutes or a single centre, and secondary confirmation was performed for doubtful samples.

## 1 Introduction

Metabolic syndrome (MetS) is a complex metabolic disorder influenced by genetic and lifestyle factors, which is a metabolic disorder including insulin resistance, abnormal glucose-lipid metabolism, centrally oriented obesity and hypertension, etc., among which central obesity and insulin resistance are the key factors of metabolic syndrome. The prevalence of metabolic syndrome is increasing year by year in different countries and regions due to complex factors such as the level of economic development, racial structure, age structure of the population, and dietary spectrum of nutritional intake, which lead to differences among populations (Qin et al., 2020; Yao et al., 2021; Ali et al., 2024; Babicki et al., 2024). The pathogenesis of metabolic syndrome includes a variety of risk factors, both inborn genetic and acquired, which are caused by pathophysiological alterations such as systemic inflammatory states, oxidative stress, and haemodynamic dysfunction (Fahed et al., 2022; Silveira et al., 2022). The metabolic syndrome has become a major health problem worldwide and is not only strongly associated with chronic diseases (cardiovascular disease, type II diabetes), but may also have an unpredictable and long-lasting adverse effect on male fertility and male genitourinary disorders. There is a strong association between MetS-related components (central obesity, hyperglycaemia, hyperlipidaemia, and hypertension) and male disorders (premature ejaculation, erectile dysfunction, and hypogonadotropic hypogonadism), which share common risk factors and similar pathophysiological mechanisms (Iafrate et al., 2022). Routine semen analysis has limitations in the diagnosis of male infertility and in assessing the effectiveness of treatments (Santi et al., 2018), thus the sperm DNA Fragmentation Index (DFI) has emerged as a new method of assessing fertility based on evidence of previous fertility, which can more accurately explain recurrent spousal miscarriage and unexplained male infertility, especially the predictive power of recurrent biochemical pregnancy or indeterminate abortion in early pregnancy. Sperm DNA integrity is critical for normal embryonic development and pregnancy outcome, and an increased sperm DFI is associated with advanced male age, varicocele, smoking and alcohol abuse and poor lifestyle (Szabó et al., 2023). Current research on sperm DFI focuses on male reproductive health, and how to reduce sperm DFI and improve pregnancy outcomes is a hot clinical topic of concern. In this study, we investigated the correlation between metabolic syndrome and sperm DFI, and developed individualised and precise metabolic management strategies through a prospective long term follow-up study in several medical institutions in Anhui, China.

## 2 Object and method

### 2.1 Study object

The study was a prospective follow-up study, and the study population was included in the management of the Male Reproductive Health Follow-up Database (2020–2024) in Bozhou City, Anhui Province, China. The study was a multi-centre clinical study including two large regional medical institutions in Bozhou region: The People’s Hospital Bozhou and Bozhou Hospital of Traditional Chinese Medicine, and several Bozhou community health institutions and specialist hospitals. In this study, 13,580 cases of men of reproductive age were screened in outpatient clinics, 1,029 cases were included in the study by the inclusion criteria and exclusion criteria, 21 cases withdrew from the study and were lost to follow-up, and 1,008 cases finally completed the study, with sperm DFI according to the reference value, and the DFI of ≤30% was considered to be integrity intact or general, and was set up as the normal group with normal DFI (Control group, 858 cases); DFI >30% is poor integrity, set as DFI abnormal group (Observation group, 150 cases). The baseline information, occupational and environmental exposure history, metabolic endocrine-related indexes, and fertility assessment-related indexes of patients in the two groups were analysed, and the patients were followed up for metabolic indexes and fertility.

### 2.2 Study data indicators

#### 2.2.1 Baseline information

Age, infertility cycle (months), household income, residence, smoking history, drinking history, sperm collection season, genitourinary inflammation, pubertal parotitis, testicular trauma, environmental exposure, occupational exposure.

Smoking history: the patient was a chronic smoker or smoked excessively during the spermatogenic cycle (3 months).

Drinking history: the patient has a history of chronic alcohol consumption or excessive alcohol consumption during the spermatogenic cycle (3 months).

#### 2.2.2 Endocrine metabolism-related indicators

Body mass index (BMI), Waist circumference (WC), Triglycerides (TG), High density lipoprotein (HDL), Blood pressure (BP), fasting blood-glucose (FBG).

#### 2.2.3 Fertility assessment indicators

Male infertility: cohabiting regular sex without contraception and infertile for more than 1 year.

Spousal adverse maternity: various types of miscarriages ≥3, history of preterm labour, perinatal mortality, birth defects, ectopic pregnancy, trophoblastic disease, previous pregnancy complications and comorbidities.

Varicocele: Ultrasound diagnostic criteria for varicocele (one of the following can be met): (1) patient standing position, internal diameter of the spermatic vein ≥2 mm at rest, and positive for reflux accompanied by Vasalva action; (2) patient standing position, internal diameter of the spermatic vein ≥ 3 mm during Vasalva action (Freeman et al., 2020).

Sexual dysfunction: refers to the individual in the libido, erection, sexual intercourse, ejaculation and sexual sensation and other aspects of the occurrence of one or more abnormal performance, divided into erectile dysfunction, premature ejaculation, ejaculation process is significantly delayed or not ejaculate at orgasm.

### 2.3 MetS diagnostic criteria

There is no international unified and clear diagnostic criteria for MetS, but different studies have basically agreed on the diagnosis of MetS, which can be diagnosed as MetS by fulfilling three out of the five items: obesity, hyperlipidaemia, hypertension, elevated blood glucose, and low high density lipoprotein (Handelsman et al., 2024; Alberti et al., 2009; Balkau and Charles, 1999; Ogawa et al., 2022; Grundy et al., 2005). Based on the reference data of metabolic indexes in the Chinese men of reproductive age group and the study design, this study supplemented some metabolic indexes and redefined the diagnostic criteria of MetS. One point was assigned for each of the following criteria, and a metabolic score of ≥3 was diagnostic of MetS.

(I) Obesity, BMI ≥28 kg/m^2^; abdominal obesity, waist circumference ≥90 cm; waist-hip ratio (waist/hip) ≥1; (II) G ≥ 1.7 mmol/L; and/or moderately severe fatty liver; and/or currently in the phase of lifestyle improvement or medication for lipid control; (III) HDL< 1.03 mmol/L, and/or are in the stage of lifestyle improvement or pharmacological treatment to raise HDL; (IV)Clinical diagnosis of hypertension; typical clinical symptoms of hypertension, and/or systolic blood pressure (SBP) ≥130 mmHg and/or diastolic blood pressure (DBP) ≥85 mmHg on 3 measurements on non-simultaneous days without the use of antihypertensive drugs; and/or Currently in the stage of improving lifestyle or medication to control blood pressure; (V) Clinical diagnosis of diabetes mellitus; typical clinical symptoms of diabetes mellitus, fasting plasma glucose concentration ≥6.1 mmol/L and 2-h postprandial plasma glucose concentration ≥7.8 mmol/L; impaired glucose tolerance (IGT); and/or currently in the phase of improving lifestyle or medication to control blood glucose.

### 2.4 Inclusion and exclusion criteria

#### 2.4.1 Inclusion criteria

(1) Men of childbearing age with reproductive needs, aged ≥20 years and ≤50 years; (2) Han Chinese; (3) Have not suffered from major family or social problems in the past 6 months; (4) Agree to be included in the study, sign the informed consent for inclusion and the notification of potential benefits or risks of the study; (5) complete outpatient or inpatient medical records and follow-up information.

#### 2.4.2 Exclusion criteria

(1) Malignant tumour, serious organic cardiovascular and cerebrovascular diseases or mental and psychological disorders; (2) Obstructive or non-obstructive azoospermia, severe oligozoospermia, which seriously affects fertility; (3) With chromosomal abnormalities or genetic hereditary diseases; (4) Family hereditary diseases.

### 2.5 Study indicators methodology

#### 2.5.1 Measurement of clinical biochemical metabolic indexes

Biochemistry laboratory of the testing centre: fully automated biochemistry tester or semi-automated biochemistry tester to determine FBG, TG, HDL by enzymatic or chemical methods.

#### 2.5.2 Sperm DNA fragmentation index

Male laboratory of the Centre for Reproductive Medicine: sperm DNA fragmentation index was determined by flow cytometry using the Fluorescence staining method.

Test Principle: Using flow cytometry on fresh or frozen semen, spermatozoa with DNA fragments are denatured by acid to form single-stranded DNA, which fluoresces red when combined with the dye acridine orange. Normal spermatozoa without DNA fragments can maintain the intact double-stranded DNA structure after acid treatment and fluoresce green when combined with acridine orange. The ratio of red to green light reflects the rate of DNA fragmentation. Fluorescent dyes fluoresce red when bound to fragmented DNA single strands and green when bound to double-stranded DNA, and a higher red-to-green ratio indicates an increased rate of DNA fragmentation (Evenson and Wixon, 2008; Evenson et al., 1999).

Sample requirements: Fresh semen samples were collected from men who abstained from sex for 2–7 days and ejaculated through masturbation. Some semen samples are kept frozen at −18°C for a period not exceeding 7 days due to environmental and equipment constraints.

Sperm DNA staining solution, Sperm DNA staining solution main components, reagent A: sperm washing solution (20x) (disodium hydrogen phosphate, sodium dihydrogen phosphate, sodium chloride); reagent B: fixation solution (sodium chloride, TritonX-100); reagent C: fluorescence staining solution (acridine orange, disodium hydrogen phosphate, sodium chloride). Anhui Anke Biological Engineering (Group) Co, No. 20160023.

Test methods: (1) take semen samples to be examined and adjust the concentration to 1-2 × 10^6^/mL with reagent A application solution; semen samples with abnormal liquefaction were processed in accordance with semen samples: semen liquefying agent for 100:1 by adding liquefying agent to liquefy them completely first; (2) take 100 μL of the adjusted concentration of semen samples in a test tube, add 200 μL of reagent B, mix well, and incubate at 0°C for 30 s; (3) Add 600 μL of reagent C, mix well; (4) Count the ratio of red-light spermatozoa to green-light spermatozoa using flow cytometry.

### 2.6 Follow-up plan

(1) Follow-up timeframe: the follow-up of this study was set at 12 months after inclusion in the study. The cut-off date for study follow-up is 31 January 2025. (2)Follow-up content: fertility assessment, sperm DFI values, metabolic scores, etc. (3) Form of follow-up: for outpatient follow-up patients, the outpatient medical record browsing system is available to collect data for study information. For patients who could not come to the hospital for follow-up, telephone call or letter questionnaire could be used.

### 2.7 Guidance for interventions

(1) Scientific weight loss programme: short-term weight loss goal, within 3–6 months, to achieve weight loss usually 10%–15% of body weight; long-term weight loss goal: the ideal maintenance of weight loss status for at least 1–2 years. (2)Daily Exercise Guidelines: 24-h exercise guidelines (including moderate to high intensity physical activity, reasonable sleep duration and reduction of sedentary time). (3) Lifestyle recommendations: Lifestyle interventions have different focuses according to different metabolic goals, with the goal of improving metabolic syndrome risk factors. (4)Dietary recipes: Time-Restricted Eating (TRE) for 8–10 h, weight loss diets, active encouragement of high quality protein intake, and control of fat and carbohydrate intake.

### 2.8 Quality control

(1) Enrolment of research subjects: Researchers were screened for enrolment based on diagnostic criteria, inclusion criteria and exclusion criteria, and data of all research subjects in this study were collected and matched by two people independently through the medical record browsing system, and then verified by the researcher (the third person) for inclusion in the standardised data collection and management system. (2) Data validation: Clinical data were reviewed and validated by a third party. (3) Data management: In order to strengthen the study’s standardisation of clinical trial data management and to improve the quality of the study and conclusions, this study used a standardised data collection and management system (FAST-Date) developed in-house, including a paper Case Record Form, Electronic Data Capture (EDC), an internet-based EDC system, and in-process quality control (QC) and real-time online QC for data collection and management throughout the entire process of the study.

### 2.9 Ethical review

This study complied with the principles of the Declaration of Helsinki, and was approved by the Ethics Committee of The People’s Hospital Bozhou, (Bo-Medical Ethics Review 2024No.146 and 2025No.75). This study was pre-registered with the China Clinical Trial Registry (ChiCTR) under process identification number: PID253645. Registration number: ChiCTR2500101473. In this study, due to limited objective conditions, some of the patients who were included in the study were enrolled by supplemental enrolment in order to fulfil the purpose of the study and the sample size.

The semen samples required for laboratory tests in the male department of reproductive medicine were taken with the informed consent of the patients, and the semen samples were all destroyed after completion of the tests and were not used for the rest of the non-reproductive medicine testing programmes. All subjects in this study signed an informed consent form for inclusion in the study and completed a fertility information questionnaire in the online survey/outpatient clinic. Subjects’ informed consent: The study used patients’ clinical information and informed consent was obtained from all subjects by telephone or in writing.

Clinical Trial Registration Note: The registration procedure and content are fully in line with the process and standards of the WHO International Clinical Trial Registration Platform (WHO ICTRP), the International Committee of Medical Journal Editors (ICMJE), and the Chinese Clinical Trial Registry (ChiCTR), and the design plan and some necessary study information will be made available to the public after the article is published. After the article is published, the design plan of the clinical trial and some necessary information of the study will be disclosed to the public, Can be contacted via email ChiCTR:chictr003@chictr.org.cn (study registration information) or corresponding author email; 8448223376@qq.com (study process information).

### 2.10 Statistical method

SPSS21.0 statistical software was used for data processing and analysis. Measurement information was tested by t-test between groups, and one-way ANOVA was used for comparison between multiple groups; count information was described by rate, and X^2^ test was used for comparison between groups; measurement information conforming to normal distribution was expressed by (‾x ± s), and t-test of independent samples was used for comparison between groups; measurement information not conforming to normal distribution was expressed by median (interquartile spacing) M (P_25_, P_75_), and non-parametric Mann-Whitney U test; count data were expressed as number of cases (percentage) [n (%)], and comparisons between groups were made using the X^2^test; correlation analyses were performed using the Spearman rank correlation coefficient. Threshold analysis was performed using the segmented package. Bootstrap sampling method was used for mediation effect test and Bayesian method was used for mediation effect value estimation.

All variables were screened by Logistic regression analysis using the forward likelihood method to screen single factors, and all variables with *P* < 0.05 in the single factor analysis were included in the multifactor Logistic regression analysis. Predictive models were constructed using the parameters with *P* < 0.05 in the multifactor analysis using the R Programming Language (4.42). Logistic regression model was constructed by dividing the study data (n = 1,008) into training set (n = 807) and test set (n = 201). Column Nomogram, Calibration Curve, Decision Curve Analysis (DCA) and Clinical Impact Curve (CIC) were plotted to assess the consistency between the predicted probability of the model and the actual probability of occurrence, and also to evaluate the best diagnostic value of the model in clinical decision-making, and to evaluate the utility and applicability of the model in clinical decision-making for the net benefit of the best diagnostic value. The test level was α = 0.05.

## 3 Results

### 3.1 Transect survey

**FIGURE 1:**
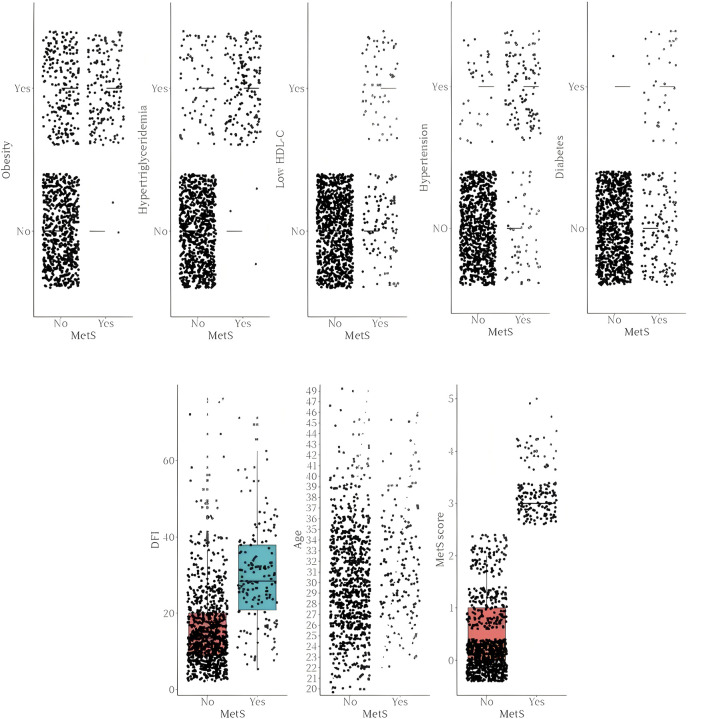
Transect survey of the metabolic syndrome.

The summary report of the cross-sectional survey showed that according to the sperm DNA fragmentation index reference range, DFI was abnormal in 150 cases (14.88%) and DFI was normal in 858 cases (85.12%). According to MetS definition and metabolic score, metabolic syndrome was 158 cases (15.67%) and non-metabolic syndrome was 850 cases (84.33%). The specific distribution of metabolic components such as obesity, hypertension, hyperlipidemia, low HDL-C, diabetes mellitus and sperm DFI and age for different groups of metabolic syndrome are detailed in Figure 1.

### 3.2 Between-group comparison of baseline information for the observation and control groups

Comparison between the groups showed that there was no statistically significant difference between the two groups in terms of baseline information such as age, smoking history, history, drinking history, household income, residence, sperm collection season, genitourinary inflammation, and sexual dysfunction (*P* > 0.05). see Table 1 for details.

**TABLE 1:** Analysis of the baseline information between the two groups.

Groups	Observation group (N = 150)	Control group (N = 858)	$\chi^{2}$	*P*
Age			1.195	0.274
≤30	66 (44.00)	419 (48.83)		
>30	84 (56.00)	439 (51.17)		
Smoking history			0.810	0.368
Yes	28 (18.67)	135 (15.73)		
No	122 (81.33)	723 (84.27)		
Drinking history			2.662	0.103
Yes	19 (12.67)	73 (8.51)		
No	131 (87.33)	785 (91.49)		
Household income			0.169	0.681
High	39 (26.00)	237 (27.62)		
Low	111 (74.00)	621 (72.38)		
Residence			0.010	1.000
City	41 (27.33)	238 (27.74)		
Villages	109 (72.67)	620 (72.26)		
Sperm collection season			1.869	0.172
Spring-summer	109 (72.67)	575 (67.02)		
Autumn-winter	41 (27.33)	283 (32.98)		
Genitourinary inflammation			0.100	0.752
Yes	21 (14.00)	112 (13.05)		
No	129 (86.00)	746 (86.95)		
Sexual dysfunction			0.004	0.949
Yes	10 (6.67)	56 (6.53)		
No	140 (93.33)	802 (93.47)		

### 3.3 Between-group comparison of MetS and metabolic scores in the observation and control groups

Intergroup comparison showed a statistically significant difference in MetS and MetS scores (*P* < 0.001). Among the observation and control groups in the proportion of metabolic syndrome was 49.33% vs. 10.03%, while the difference in MetS scores was also more significant. See Table 2 for details.

**TABLE 2:** Analysis of MetS and metabolic scores in the two groups n (%).

Groups	Observation group (N = 150)	Control group (N = 858)	$\chi^{2}$ /*F*	*P*
MetS			151.045	<0.001
Yes	74 (49.33)	84 (10.03)		
No	76 (50.67)	774 (89.97)		
MetS scores			188.316	<0.001
0	36 (24.00)	580 (67.60)		
1	23 (15.33)	130 (15.15)		
2	17 (11.33)	64 (7.46)		
3	51 (34.00)	74 (8.63)		
4	22 (14.67)	9 (1.05)		
5	1 (0.67)	1 (0.11)		

### 3.4 Comparison of age and infertility cycle between different groups of MetS and sperm DFI

According to the definition of MetS, the study subjects were divided into two groups of MetS/non-MetS. According to the reference range of sperm DNA fragmentation index, they were divided into three groups, <15, 15–30, and >30, and the differences between age and infertility cycle were compared between the groups. The results showed that there was statistical significance in comparing age between the two groups of MetS/non-MetS, and there was statistical significance in comparing infertility cycle (*P* < 0.05). There was no statistical significance in comparing age between the three groups of DFI, and there was also no statistical significance in comparing two by two between the groups (*P* > 0.05); there was no statistical significance in comparing infertility cycle (*P* > 0.05) but the differences in the DFI <15 and DFI>30 were statistically significant (*P* = 0.029), see Table 3 for details.

**TABLE 3:** Comparative analysis of age and infertility cycle between different subgroups of MetS/non-MetS and sperm DFI.

Groups	n (%)	Age(Y)	t/F	*P*	Infertility cycle (M)	t/F	*P*
MetS			−4.216	<0.001		−2.682	0.013
Yes	158 (15.67)	30.04 ± 4.98			11.64 ± 3.91		
No	850 (84.33)	31.87 ± 5.18			16.15 ± 4.81		
DFI			2.568	0.077		2.684	0.089
<15	507 (50.30)	29.97 ± 4.92			11.60 ± 4.45		
15–30	351 (34.82)	30.66 ± 5.23			13.78 ± 4.32		
>30	150 (14.88)	30.75 ± 5.04			16.62 ± 4.98		

### 3.5 Correlation analysis between MetS score and sperm DFI, values.

To further explore the correlation between Met scores and DFI values, one-way ANOVA showed that the difference between DFI and MetS scores was statistically significant (*P* = 0.021), and SNK two-by-two comparisons were performed to show that the difference was statistically significant in comparisons between groups with scores of 0, 1, 2, 3, and 4 (*P* < 0.05). Due to the small amount of data in subgroup 5 (n = 2), it was not included in the statistics. See Table 4 for details.

**TABLE 4:** Correlation analysis between Met scores and DFI values.

MetS score	0	1	2	3	4	5	F	*P*-T
DFI	14.14 ± 9.53	19.56 ± 11.77	22.41 ± 10.17	28.69 ± 12.83	36.37 ± 14.65	-	63.918	<0.001

**FIGURE 2:**
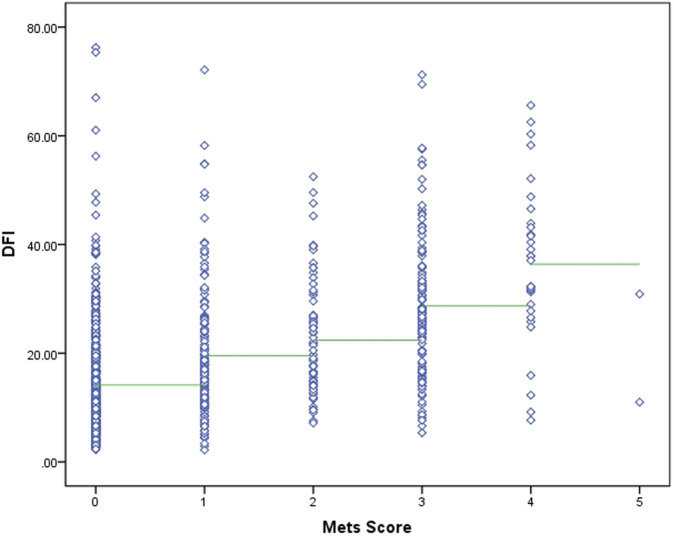
Correlation analysis between metabolic scores and DFI values.

The two sets of variables in this study, in terms of data characteristics, metabolic scores are multicategorical ordered rank information and sperm DFI is a continuous variable, and correlation analyses were performed using Spearman’s rank correlation coefficient. After Spearman correlation analysis, there was a medium-strength positive correlation between metabolic score and sperm DFI (r = 0.475, *P* < 0.001). See Figure 2 for details.

### 3.6 Analysis of the relationship between MetS and sperm DFI thresholds

**FIGURE 3:**
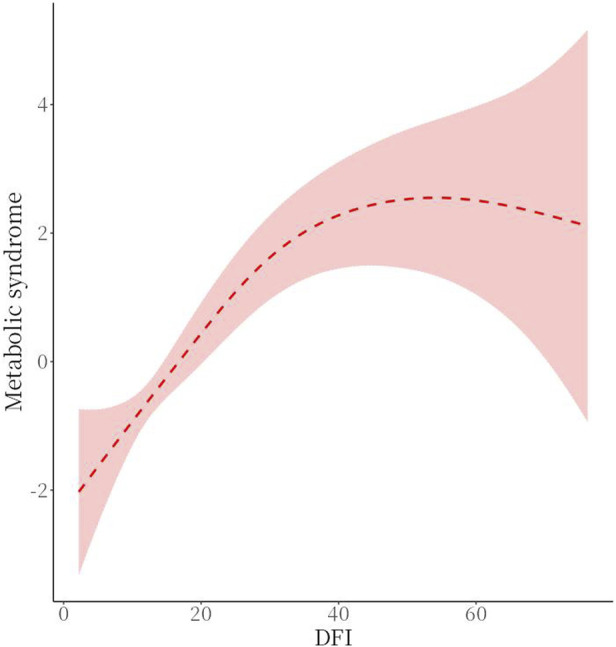
Smoothed graphs of MetS and sperm DFI (dose-response relationship plots).

There was a threshold effect (*P* for likelihood test <0.001) for the association of sperm DFI and MetS, which was positive overall [OR (95%CI): 1.09 (1.07–1.11)], when DFI <32.26 [OR (95%CI): 1.15 (1.12–1.19)], and when DFI ≥32.26, no association between DFI and MetS was found. See Table 5 and Figure 3 for details.

**TABLE 5:** MetS in relation to sperm DNA fragmentation index thresholds.

Outcome	Effect	*P*
Model 1 Fitting model by standard linear regression	1.09 (1.07–1.11)	<0.001
Model 2 Fitting model by two-piecewise linear regression		
Inflection point	32.26	
<32.26	1.15 (1.12–1.19)	<0.001
≥32.26	1.01 (0.97–1.04)	0.733
*P* for likelihood test		<0.001

### 3.7 Subgroup analysis of the association between MetS and adverse maternity outcomes in spouses

**FIGURE 4:**
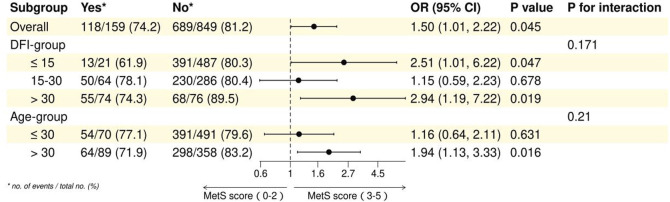
Forest plot of subgroup analysis of association between MetS and spousal adverse maternity outcomes.

In the overall analysis, the association between MetS and adverse maternity outcomes was statistically significant (OR = 1.50, 95% CI: 1.01–2.22, *P* = 0.045). In the DFI subgroup, the association of metabolic syndrome with adverse pregnancy outcomes was significant in both ≤15 and >30 (≤15: OR = 2.51, 95% CI: 1.01–6.22, *P* = 0.047; >30: OR = 2.94, 95% CI: 1.19–7.22, *P* = 0.019), whereas in 15–30 there was no significant association (OR = 1.15, 95% CI: 0.59–2.23, *P* = 0.678). In subgroup analyses, the DFI subgroup interaction was not statistically significant (*P* for interaction = 0.171). Age subgroup analyses showed a significant association between metabolic syndrome and adverse pregnancy outcomes in those aged >30 years (OR = 1.94, 95% CI: 1.13–3.33, *P* = 0.016), whereas there was no significant association in those aged ≤30 years (OR = 1.16, 95% CI: 0.64–2.11, *P* = 0.631), but the age subgroup interaction was not statistically significant (*P* for interaction = 0.21). These findings highlight the differential impact of MetS on adverse maternity outcomes across distinct subgroups. See Table 6 and Figure 4 for details.

**TABLE 6:** Subgroup analysis of the association between MetS and spousal adverse maternity outcomes in spouses (univariate logistic model).

Subgroup	Yes^a^	No^a^	Crude OR (95% CI)	*P* Value	*P* For interaction
Overall	118/159 (74.2)	689/849 (81.2)	1.50 (1.01–2.22)	0.045	
DFI-group					0.171
≤15	13/21 (61.9)	391/487 (80.3)	2.51 (1.01–6.22)	0.047	
15–30	50/64 (78.1)	230/286 (80.4)	1.15 (0.59–2.23)	0.678	
>30	55/74 (74.3)	68/76 (89.5)	2.94 (1.19–7.22)	0.019	
Age-group					0.21
≤30	54/70 (77.1)	391/491 (79.6)	1.16 (0.64–2.11)	0.631	
>30	64/89 (71.9)	298/358 (83.2)	1.94 (1.13–3.33)	0.016	

^a^ no. of events/total no. (%).

### 3.8 Unifactorial-multifactorial logistic analyses affecting sperm DFI

All clinical and laboratory factors that may produce abnormalities in sperm DNA fragmentation index were included in one-way logistic regression analysis, which showed that pubertal parotitis, environmental exposure, varicocele, metabolic syndrome, obesity, hyperlipidaemia, Low-HDL, hypertension and diabetes mellitus were statistically significant (*P* < 0.05), while the differences in other indices were not statistically significant (*P* > 0.05). Variables with *P* < 0.05 in the univariate analysis were included in the multifactorial logistic regression model, which showed that varicocele (OR = 1.975), obesity (OR = 2.296), hyperlipidaemia (OR = 2.422), and Low-HDL (OR = 3.654) were the independent risk factors for the abnormal sperm DNA fragmentation indices, in which the obese patients risk was 2.296 times higher than that of non-obese patients, hyperlipidaemic patients had 2.422 times higher risk than that of non-hyperlipidaemic patients, and low-HDL patients had 3.654 times higher risk than that of H-HDL patients. See Table 7 for details.

**TABLE 7:** Univariate analysis and multi-factor logistic regression analysis.

Variable	Single factor logistic regression analysis		Multivariate logistic regression analysis	
	OR (95%CI)	*P*	OR (95%CI)	*P*
Smoking	1.229 (0.784, 1.928)	0.369	—	—
Drinking	1.560 (0.911, 2.670)	0.105	—	—
Residence	0.980 (0.664,1.446)	0.918	—	—
Household income	0.921 (0.621, 1.366)	0.681	—	—
Sperm collection season	1.308 (0.889, 1.925)	0.172	—	—
Genitourinary inflammation	1.084 (0.656, 1.792)	0.752	—	—
Pubertal parotitis	3.208 (1.168,8.811)	0.024	1.057 (0.307, 3.636)	0.930
Testicular trauma	2.168 (0.569, 8.268)	0.257	-	-
Environmental exposure	2.045 (1.036, 4.036)	0.039	1.805 (0.798, 4.082)	0.156
Occupational exposure	1.892 (0.874, 4.094)	0.105	—	—
Varicocele	2.407 (1.544, 3.751)	<0.001	1.975 (1.190, 3.279)	0.008
Sexual dysfunction	1.023 (0.510, 2.053)	0.949	—	—
Metabolic syndrome	8.855 (5.991, 13.087)	<0.001	0.949 (0.365, 2.472)	0.915
Obesity	6.395 (4.329, 9.448)	<0.001	2.296 (1.342, 3.929)	0.002
Hyperlipidaemia	8.025 (5.516, 11.674)	<0.001	2.422 (1.226, 4.786)	0.011
Low-HDL	11.465 (6.588, 20.042)	<0.001	3.654 (1.688, 7.910)	0.001
Hypertension	5.012 (3.406, 7.643)	<0.001	1.851 (0.952, 3.598)	0.069
Diabetes	4.429 (2.228, 8.084)	<0.001	1.327 (0.553, 3.188)	0.526

### 3.9 Analysis of sperm DFI mediation in MetS and spousal adverse maternity

The exposure factors were Obesity and Hyperlipidaemia in the Metabolic Syndrome component, the outcome was Spousal Adverse Maternity, and the mediator was Sperm DFI. mediation effect tests were conducted using Bootstrap sampling method, and mediation effect value estimation method Bayesian method. The mediator analysis pathway results showed that Obesity and Hypertriglyceridemia lead to abnormal sperm DFI and also to adverse maternity, but it has not been proved that sperm DFI leads to adverse maternity. See Table 8 for details.

**TABLE 8:** MetS and sperm DFI mediated pathways of analysis of spousal adverse maternity.

Path	Relation	SE	Lower	Upper	*P*	β (95%CI)
Obesity -- > Adverse Maternity	expose-- > outcome	0.18	0.15	0.84	<0.01	0.50 (0.15 ∼ 0.84)
DFI -- > Adverse Maternity	medium-- > outcome	0.01	−0.03	0.001	0.13	−0.01 (−0.03 ∼ −0.001)
Hypertriglyceridemia -- > DFI	expose-- > medium	0.84	10.85	14.12	<0.01	12.48 (10.85 ∼ 14.12)
Hypertriglyceridemia -- > Adverse Maternity	expose-- > outcome	0.20	0.11	0.91	0.01	0.51 (0.11 ∼ 0.91)
DFI -- > Adverse Maternity	medium-- > outcome	0.01	−0.03	0.001	0.16	−0.01 (−0.03 ∼ −0.001)

### 3.10 Comparison of sperm DFI and metabolic score before and after intervention

Taking the intervention guidance, the comparison between the control group and the observation group regarding the sperm DNA fragmentation index and metabolic score before and after the intervention was as follows: the comparison of sperm DFI score and metabolic score before and after the intervention in the observation group was statistically significant (*P* < 0.001); the comparison of sperm DFI score and metabolic score before and after the intervention in the control group was not statistically significant (*P* > 0.05); the comparison of the observation group and the control group before and after the intervention at different nodes sperm DFI score and metabolic score were statistically significant when compared between the two groups (*P* < 0.001). There was a significant reduction in sperm DFI score and metabolic score in the observation group compared to the control group after the intervention. See Table 9 for details.

**TABLE 9:** Comparison of sperm DFI and metabolic scores before and after interventionx.

Groups	N	Sperm DFI		t	*P*	Metabolic score		Z	*P*
		Pre-inter	Post-inter			Pre-inter	Post-inter		
Observation group	150	40.68 ± 10.39	27.14 ± 15.79	6.038	<0.001	1 (0,3)	1 (0,2)	−4.944	<0.001
Control group	858	14.18 ± 7.01	17.78 ± 10.37	0.639	0.525	0 (0,2)	0 (0,2)	−1.414	0.157
t/Z		−30.06	−3.913			−6.050	−4.494		
*P*		<0.001	<0.001			<0.001	<0.001		

### 3.11 Predictive modelling of DFI affecting spermatozoa

**FIGURE 5:**
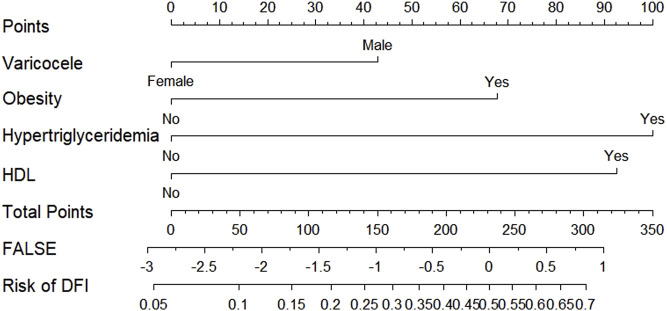
Nomogram of the risk of abnormal sperm DFI.

**FIGURE 6:**
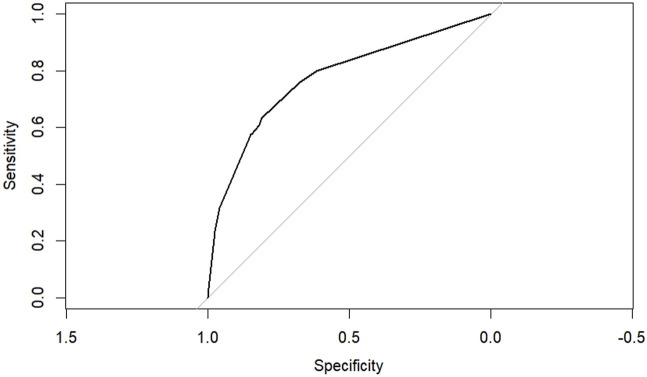
ROC curve of the training set.

**FIGURE 7:**
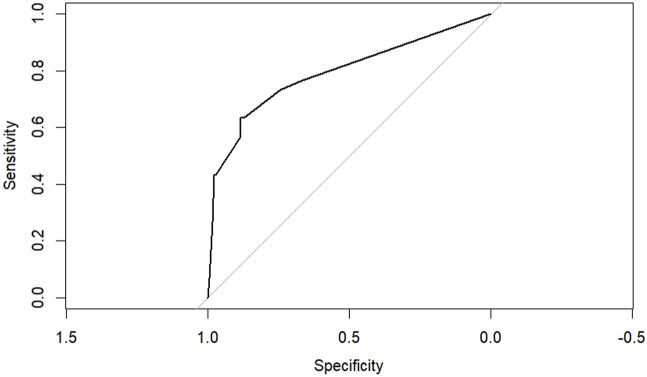
ROC curve of the test set.

**FIGURE 8:**
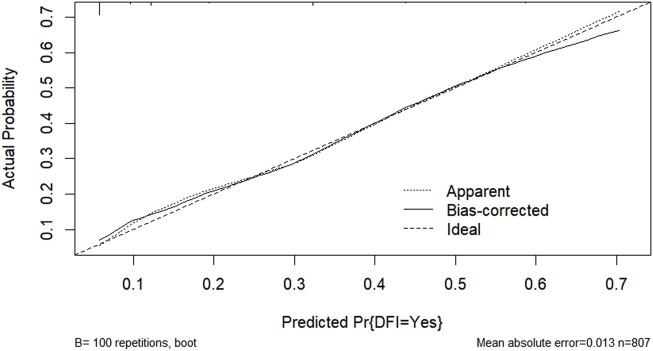
Predicting training set calibration curves for column line graph models.

**FIGURE 9:**
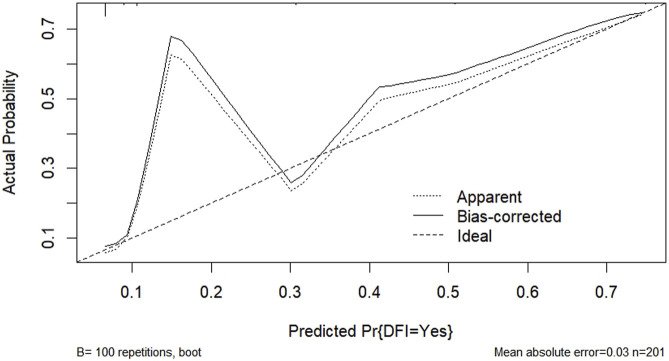
Predicting test set calibration curves for column-line diagram models.

According to the results of multifactorial logistic regression analysis, the four factors of statistically significant indicators Varicocele, Obesity, Hyperlipidaemia and Low HDL were used as predictors, and sperm DFI was used as the ending variable, and the nomogram was plotted using the R software, as detailed in Figure 5. The Bootstrap self-sampling method (1,000 times of self-sampling) was used to carry out the internal validation, divided into training set and test set, training set. The training set AUC value of the predictive model was calculated as 0.77 (95% CI: 0.73–0.82) and the test set AUC value was 0.79 (95% CI: 0.69–0.89) by depicting the receiver operating characteristic (ROC) curve, indicating that the model has good predictive efficacy, as shown in Figures 6, 7. The calibration curves for the training set showed an absolute error of 0.013, and the calibration curves for the test set showed an absolute error of 0.03, which is in good agreement, as shown in Figures 8, 9.

**FIGURE 10:**
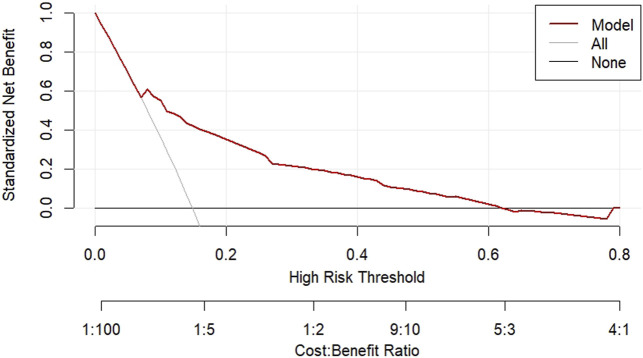
DCA decision plot for the training set.

**FIGURE 11:**
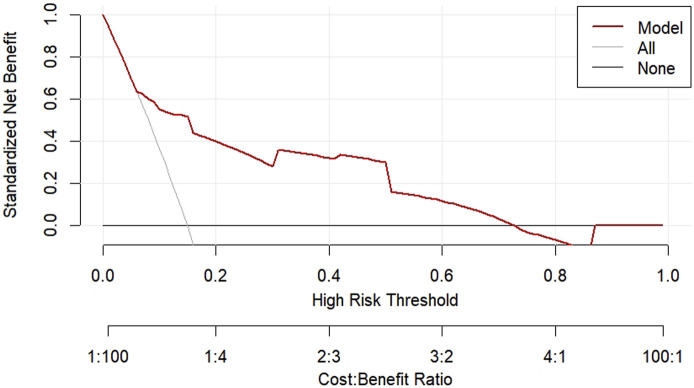
DCA decision plot for the test set.

**FIGURE 12:**
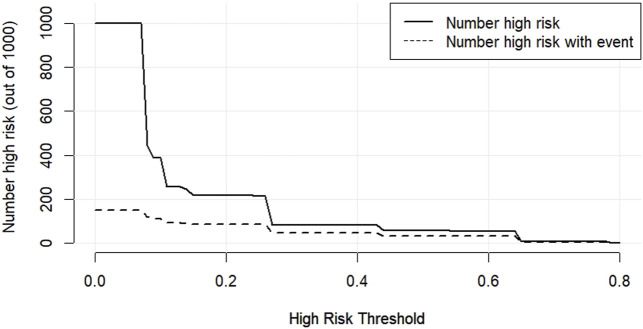
CIC for the training set.

**FIGURE 13:**
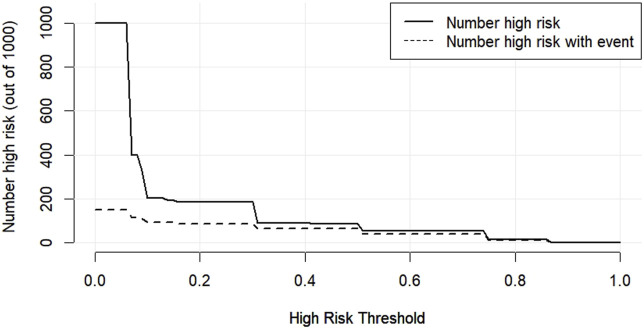
CIC for the test set.

The DCA decision curve analysis showed that the use of a line graph to predict sperm DFI abnormalities provided a net clinical benefit when the training set threshold probability ranged between approximately 6% and 62% and the test set threshold probability ranged between approximately 8% and 72%. See Figures 10, 11 for details. The CIC analysis showed the clinical validity of the prediction model. In both the training and test sets, when the threshold probability >30% prediction score probability value, the prediction model determined that the population at high risk of sperm DFI abnormality was highly matched with the population that actually occurred sperm DFI abnormality, confirming the high clinical efficiency of the prediction model,as shown in Figures 12, 13.

## 4 Discussion

Metabolic syndrome has become an increasing public health concern as people’s modern lifestyles change. From 1999 to 2014, the overall trend of MetS prevalence increased in the United States (from 27.6% to 32.3%) (Li et al., 2023). In the Chinese population aged 15 years and older, the pooled prevalence was 24.5% (95% CI: 22.0%–26.9%), 19.2% (95% CI: 16.9%–21.6%) in males and 27.0% (95% CI: 23.5%–30.5%) in females, and the overall prevalence of MetS increased with age, with those living in urban areas being Individuals were more likely to develop MetS than those living in rural areas, and hypertension was the most prevalent component of MetS in men (52.8%) (Li et al., 2016).

The intergroup comparison between the observation and control groups in this study showed that there was no statistically significant difference between the two groups in terms of age, smoking history, drinking history, household income, residence, sperm collection season, genitourinary inflammation and sexual dysfunction (*P* > 0.05). However, intergroup comparison of MetS and metabolic scores between the two groups showed a statistical difference (*P* < 0.001). One-way ANOVA showed statistically significant differences between sperm DFI and different MetS score values (*P* = 0.021), two-by-two comparisons showed statistically significant differences in comparisons between groups with scores of 0, 1, 2, 3, and 4 (*P* < 0.05), and there was a moderate-strength positive correlation between metabolic scores and DFI as analysed by Spearman’s correlation (r = 0.475. *P* < 0.001). Therefore, it can be concluded that there is a positive correlation between metabolic syndrome and metabolic score and sperm DFI values. Threshold analysis of the association between sperm DFI and MetS was then performed, and the results showed that there was threshold effect between the two (*P* < 0.001), and that overall sperm DFI and MetS were positively correlated [OR (95%CI): 1.09 (1.07–1.11)], which was specifically shown in that when the sperm DFI was ≤32.26, the DFI and MetS were positively correlated [OR (95%CI): 1.15 (1.12–1.19)], while no association was found between sperm DFI and MetS when sperm DFI >32.26. This result suggests that when sperm DFI is too high (>32.26), it is often the result of other non-metabolic direct factors such as older age and varicocele (Szabó et al., 2023; Zhang et al., 2022; Lira et al., 2024). Therefore when sperm DFI is too high in men, clinical attention often needs to be focused on non-metabolic factors rather than metabolic factors in the traditional sense, which is also consistent with clinical experience.

According to the definition of MetS, the study subjects were divided into two groups of MetS/non-MetS, and at the same time, according to the reference range of sperm DNA fragmentation index, they were divided into three groups of <15, 15–30, and >30, and the differences between age and infertility cycle were compared among the groups. There was a statistically significant difference in the comparison of age and infertility cycle between the two groups of MetS/non-MetS (*P* < 0.05). The results show that the MetS group compared to the non-MetS group is younger, but the infertility cycle is significantly longer, the researcher believes that the reason for this unreasonable difference is that the study subjects for men of childbearing age, abdominal obesity and hypertriglyceridemia accounted for more, this trend in the age of relatively young couples group is more common, and a short period of time after marriage is easy to form ‘co-obesity’ (Cobb et al., 2016). Sperm DFI age comparison between the three groups were not statistically significant, and the comparison between the two groups was not statistically significant (*P* > 0.05). Age is generally considered to be a known significant factor in the differences in sperm DFI, with older age (>45 years) having high sperm DFI and low high DNA stainability (HDS) compared to men of other age groups (Deenadayal et al., 2020). Comparisons between the three groups of infertility cycle were not statistically significant (*P* > 0.05), but comparisons between the two groups of DFI <15 and DFI >30 were statistically significant (*P* = 0.029), indicating that sperm DFI may be relatively shorter in the infertility cycle when compared to the sperm DFI population, and that the threshold setting of sperm DFI for the infertility cycle (DFI 15–30) needs to be verified by further large-sample studies. There was a significant negative correlation between sperm DFI and sperm viability and concentration (Yang et al., 2024; Liu et al., 2023). Significantly lower sperm counts, lower concentration, viability and abnormal morphology correlate with H-DFI, thereby prolonging the infertility cycle to a certain extent. Possible reasons for the reduced fertilisation rate in patients with H-DFI include loss of sperm AKAP4/PKARII proteins, impeded sperm capacitation, and a reduced incidence of the acrosome reaction (Zhang et al., 2024).

In the overall analysis, the association of metabolic syndrome with adverse pregnancy outcomes was statistically significant (OR = 1.50,95%CI:1.01–2.22, *P* = 0.045). In subgroup analyses, the DFI subgroup interaction was not statistically significant (*P* = 0.171.) In the DFI subgroups, the association of metabolic syndrome with adverse pregnancy outcomes was significant in both ≤15 and >30 (≤15: OR = 2.51, 95% CI: 1.01–6.22, *P* = 0.047; >30: OR = 2.94, 95% CI: 1.19–7.22, *P* = 0.019), while there was no significant association in the 15–30 group (OR = 1.15, 95% CI: 0.59–2.23, *P* = 0.678). Age subgroup analysis showed a significant association between metabolic syndrome and adverse pregnancy outcomes in the age >30 group (OR = 1.94, 95% CI: 1.13–3.33, *P* = 0.016), whereas there was no significant association in the age ≤30 group (OR = 1.16, 95% CI: 0.64–2.11, *P* = 0.631), but the age subgroup interaction was not statistically significant (*P* = 0.21). These findings highlight the differential impact of metabolic syndrome on adverse maternal outcomes across age and sperm DFI subgroups. There is some potential correlation between sperm DNA fragmentation not only with male infertility, but also with adverse reproductive outcomes. The effect of metabolic syndrome components on reproductive outcomes was further investigated with sperm DFI as a mediator. The exposure factors were obesity and hyperlipidaemia in the metabolic syndrome components, the outcome was spousal adverse maternity, and the mediating factor was sperm DFI. The results of the mediation analysis pathway showed that obesity and hypertriglyceridemia led to abnormal sperm DFI, as well as the occurrence of adverse maternity, but it has not been proven that sperm DFI causes adverse maternity. Sperm DFI is higher in male partners of couples with idiopathic recurrent pregnancy loss (iRPL) (Irani et al., 2024; Zhu et al., 2020). Higher sperm DFI values are associated with higher rates of embryo (haploid) aneuploidy and significantly lower pregnancy and live birth rates (Stavros et al., 2024).

MetS is a complex and highly prevalent disorder that is associated with several known and unknown factors that may affect male infertility. Although the relationship between metabolic syndrome, metabolic components and male infertility as well as the exact mechanisms are still not fully understood, it is now widely accepted that metabolic syndrome and metabolic components, i.e., obesity, diabetes mellitus, and hyperinsulinaemia, hyperlipidaemia, etc., have a non-negligible negative impact on the male reproductive system. The present study confirmed that varicocele (OR = 1.975), obesity (OR = 2.296), hyperlipidaemia (OR = 2.422), Low-HDL (OR = 3.654) were independent risk factors for abnormal sperm DNA fragmentation indices, where obese patients were 2.296 times more at risk than non-obese patients, hyperlipidaemic patients were 2.422 times more at risk than non-hyperlipidaemic patients and patients with low HDL were 3.654 times more at risk than patients with high HDL. Central obesity is also thought to be an initiating step in the development of MetS. Obesity increases adipose tissue (AT), which leads to increased production of pro-inflammatory cytokines and adipokines, therefore inducing a systemic low-level inflammatory response, leading to MetS, insulin resistance (IR) and T_2_DM (Varra et al., 2024). At the level of sperm quality ranging from sperm viability parameters to sperm DNA integrity, obesity has been shown to be potentially negatively correlated with offspring live births, both in the natural and assisted reproduction routes (Service et al., 2023). Individuals categorised as overweight/obese showed more pronounced changes in sperm parameters and sperm DFI in response to changes in environmental exposure temperatures compared to individuals with normal BMI (Wang et al., 2024). However, the conclusion that obesity impairs semen parameters and male sperm quality, natural pregnancy, or the ability of healthy offspring in assisted reproduction is somewhat debated in different studies. Possibly due to the complex pathophysiology and interactions between gonadotropins and end organs, as well as genetic and epigenetic variations and differences on male fertility, these different aspects have led to heterogeneity of study participants and different effects on assisted reproduction outcomes and offspring health (Ameratunga et al., 2023). Obesity is frequently associated with high blood insulin concentrations, which are also associated with hyperactivation of intracellular transduction pathways. The underlying chronic hyperinsulinaemia resulting from disorders of glucose and lipid metabolism is also a driving force behind the increased activation of the hypothalamic-pituitary-gonadal (HPG) axis in patients with the metabolic syndrome, leading to a state of ‘functional hypercortisolism’. This ‘functional hypercortisolism’ prevents hypoglycaemia by antagonising the action of insulin. It also disrupts metabolic homeostasis by transferring energy from muscle to abdominal fat stores. The synergistic effect of hyperinsulinaemia and ‘functional hypercortisolism’ promotes abdominal obesity and insulin resistance, which are core pathological/physiological components of the metabolic syndrome. Hyperinsulinaemia-induced activation of the HPG axis may play an important role in the development of the metabolic syndrome, and persistent chronic hyperinsulinaemic states lead to activation of the HPG axis, which may also play an important role in spermatogenesis and maturation.

Sperm DNA fragmentation refers to spermatozoa that have been affected by adverse factors (e.g., oxidative stress, smoking, high temperature, drugs, etc.) during the formation process, so that the integrity of sperm DNA is disrupted and broken into single-stranded DNA fragments. Damage to sperm DNA may occur at two main stages: (1) In the testis, sperm DNA undergoes repair disorders during the packaging and concentration stage. (2) Within the epididymis, a range of factors such as inflammatory factor stimulation, metabolite accumulation, elevated temperature, and reactive oxygen species (ROS) can lead to epididymal hypoplasia. Sperm DFI abnormalities are associated with potentially treatable conditions including varicocele, genitourinary tract and gonadal infections, thus prompting their use as a means of improving sperm DNA integrity and reproductive outcomes. Treating underlying and ameliorable male infertility factors appears to be a promising approach to effectively reducing DFI and increasing the likelihood of achieving natural and assisted conception. These include microscopic varicocele ligation, medications such as coenzyme Q10 and vitamin E for antioxidant therapy, and a healthy and regular lifestyle with cessation of smoking and alcohol restriction may help to reduce sperm DFI (Soetandar et al., 2022; Humaidan et al., 2022). In male patients with high sperm DFI undergoing assisted reproductive technology, performing intracytoplasmic sperm injection (ICSI) using testicular sperm is superior to sperm ejaculated via masturbation and may improve clinical pregnancy rates (Esteves et al., 2020). This may be due to the fact that testicular spermatozoa have higher DNA integrity, are less affected by the external environment, and do not pass through potentially inflamed areas of the spermatic tract such as the vas deferens, seminal vesicles, and ejaculatory ducts, resulting in a lower sperm DFI, but with much less viability and concentration than masturbated ejaculated spermatozoa.

In this study, the proportion of obesity (36.51%), hypertriglyceridemia (21.33%), Low-HDL (5.65%), hypertension (13.10%), and abnormal blood glucose (3.57%). After the intervention, for the group with abnormal sperm DFI, both sperm DFI and metabolic scores were significantly lower than before the intervention (*P* < 0.001). Traditional non-pharmacological treatments for metabolic syndrome include dietary modification, exercise management, psychological support and counselling, and other health behaviours, while pharmacological treatments are mainly used for preventing or treating complications, such as adjusting blood pressure, lowering blood glucose, and lowering cholesterol. Based on the concept of tertiary prevention, clinical rehabilitation can be effectively promoted to minimise the impact of metabolic syndrome on individual and group health. The objectives of MetS health education can be divided into general objectives and phased objectives according to the time point. The general objectives: according to the differences in metabolic scores, to improve the metabolic health awareness and MetS disease management ability of people with high risk of MetS, to lower metabolic scores, to improve the quality of life, to prevent or curb the occurrence and progression of MetS, and to reduce the MetS-related diseases and complications (cardiovascular diseases, malignant tumours and other diseases), and reduce the incidence and death rate. Milestones: According to the timeliness of the goals, they are divided into short-term and long-term goals, as shown in Table 10.

**TABLE 10:** MetS-related milestones for different populations.

Target population	Metabolic score	Short-term goal	Long-term goal
Healthy population	0	(1) Continue to live a healthy lifestyle (2) Continue to stay away from risk factors	Health maintenance
High risk population	1–2	(1) Improve knowledge of metabolic syndrome (2) Improve self-management skills (3) Reduce metabolic scores	Reduced incidence of MetS
Populations with mild Mets	3–4	(1) Improve knowledge of metabolic syndrome (2) Improvement of disease self-management (3) Improving metabolic indicators (4) Improve treatment compliance	Slowing disease progression and reducing associated complications
Populations with severe Mets	5	(1) Enhance daily management of metabolic indicators (2) Enhancement of disease self-management (3) Enhancing treatment adherence (4) Improve the quality of life	Reducing deaths from MetS-related diseases

Future research needs to explore the potential mechanisms and synergies linking metabolic factors to male fertility, especially for the processes of sperm production, maturation and storage. However, given that semen quality is often a priority in the assessment of fertility in men of reproductive age, and that sperm concentration, sperm viability, total sperm count, and sperm DNA integrity are inextricably linked to semen quality in men, complications associated with metabolic syndrome may indirectly or indirectly affect male fertility, reducing the chances of spontaneous pregnancies and prolonging the years of infertility. Early education for metabolic syndrome is therefore necessary, and a healthy lifestyle is also important in the prevention and management of metabolic syndrome; encouraging moderate aerobic exercise, maintaining a sensible diet, consuming plenty of fresh vegetables and fruits, and avoiding harmful behaviours such as smoking or alcohol consumption are essential to reduce the risk of metabolic syndrome and its associated cardiovascular complications (Łakoma et al., 2023). Time-restricted eating (TRE) restricting daily dietary intake to 8–10 h without enforcing a reduction in calorie intake may help to improve glycolipid metabolism and reduce the probability of cardiovascular disease and metabolic-related complications (Manoogian et al., 2024). There is growing evidence of a link between dietary patterns and fertility. Reduced energy expenditure from daily exercise activities, increased consumption of high trans-fat and high calorie and high glycaemic index foods, and reduced intake of dietary fibre negatively impact fertility. Evaluating the impact of preconception diet on clinical fertility and reproductive outcomes in men encourages more research exploring whole diets rather than single foods or nutrients in the context of male fertility, and increasing fish and dairy and reducing processed meats can improve sperm quality (Tully et al., 2024).

This study systematically and thoroughly investigated the correlation between metabolic syndrome and sperm DFI in men of reproductive age, as well as the influencing factors of different reproductive outcomes, so as to identify a series of individualised metabolic management strategies, clarify the components of metabolic syndrome and the complex relationship between metabolic scores and sperm DFI, which can be used as a comprehensive reference standard for assessing male fertility.

## Data Availability

The datasets presented in this study can be found in online repositories. The names of the repository/repositories and accession number(s) can be found in the article/supplementary material.
